# Genome Characteristics of a Novel Phage from *Bacillus thuringiensis* Showing High Similarity with Phage from *Bacillus cereus*

**DOI:** 10.1371/journal.pone.0037557

**Published:** 2012-05-23

**Authors:** Yihui Yuan, Meiying Gao, Dandan Wu, Pengming Liu, Yan Wu

**Affiliations:** Key Laboratory of Agricultural and Environmental Microbiology, Wuhan Institute of Virology, Chinese Academy of Sciences, Wuhan, People's Republic of China; Loyola University Medical Center, United States of America

## Abstract

*Bacillus thuringiensis* is an important entomopathogenic bacterium belongs to the *Bacillus cereus* group, which also includes *B. anthracis* and *B. cereus*. Several genomes of phages originating from this group had been sequenced, but no genome of *Siphoviridae* phage from *B. thuringiensis* has been reported. We recently sequenced and analyzed the genome of a novel phage, BtCS33, from a *B. thuringiensis* strain, subsp. *kurstaki* CS33, and compared the gneome of this phage to other phages of the *B. cereus* group. BtCS33 was the first *Siphoviridae* phage among the sequenced *B. thuringiensis* phages. It produced small, turbid plaques on bacterial plates and had a narrow host range. BtCS33 possessed a linear, double-stranded DNA genome of 41,992 bp with 57 putative open reading frames (ORFs). It had a typical genome structure consisting of three modules: the “late” region, the “lysogeny-lysis” region and the “early” region. BtCS33 exhibited high similarity with several phages, *B. cereus* phage Wβ and some variants of Wβ, in genome organization and the amino acid sequences of structural proteins. There were two ORFs, ORF22 and ORF35, in the genome of BtCS33 that were also found in the genomes of *B. cereus* phage Wβ and may be involved in regulating sporulation of the host cell. Based on these observations and analysis of phylogenetic trees, we deduced that *B. thuringiensis* phage BtCS33 and *B. cereus* phage Wβ may have a common distant ancestor.

## Introduction

*Bacillus thuringiensis* (Bt) is a Gram-positive entomopathogenic bacterium belonging to the *B. cereus* group. This group includes six very closely related species: *B. cereus*, *B. anthracis*, *B. thuringiensis*, *B. mycoides*, *B. pseudomycoides*, and *B. weihenstephanensis* [1], [2]. Based on multilocus enzyme electrophoresis (MEE) data [3] and DNA sequence variations of the 16S–23S internal transcribed spacers [4], *B. thuringiensis*, *B. anthracis* and *B. cereus sensu stricto* are considered as members of a single species, *B. cereus sensu lato*.

As an important biological pesticide, *B. thuringiensis* (Bt) has been widely used for biocontrol of insect pests for several decades. During their sporulation, Bt strains produce insecticidal crystal proteins (ICPs), which are highly toxic to larvae of numerous Lepidoptera, Diptera and Coleoptera species, but are harmless to human and vertebrates [5], [6]. About 83% of Bt strains contain lysogenic phage. During Bt fermentation, lysogenic phages can caused failures in 15%–30% of the batches, resulting in severe losses [7]. Studies to resolve this problem found that chitosan oligomer and derivatives can inactivate Bt phage 1–97A partices of and inhibit its infection [8], [9]. However, the exact mechanism involved in this process is still unclear. To figure out the lysogeny control mechanism and reduce losses during Bt fermentation, more genetic information from Bt phages is needed.

At present, five genomes of phages originating from *B. thuringiensis*, have been completely sequenced, namely Bam35c [10], GIL01 [11], GIL16c [12], 0305phi8-36 [13] and MZTP-02 [7]. Phage Bam35c, GIL01 and GIL16c are all *Tectiviral* phages with high sequence identity and genomes about 15 kb in size. Bam35c is different from GIL01 by only 11 bp, while GIL16c has 83.6% of sequence identity with GIL01 [12], [14]. Phage MZTP02, isolated from a Bt subsp. *kurstaki* strain, is a tailed phage with 15,717 bp genome with 40 bp inverted terminal repeats [7]. Phage 0305phi8-36, which has 218,948 bp genome with low homology to other sequenced phages, is a atypical *Myovirus* phage [13], [15]. Based on genome analysis, Hardies *et al* classified 0305phi8-36 as a novel ancient phage lineage [15].

Besides the five Bt phages, one *B. anthracis* (Ba) phages and five *B. cereus* (Bc) phages have been sequence. Thus far, comparisons of phages have focused primarily on isolates that share the same host species. For example, five Bc phages were compared with each other, and four of them (Wβ, Gamma, Cherry and Fah) were closely related [16], [17]. Comparison of phages from different but closely related hosts can provide more information. Ba phage AP50 was found to be closely related to Bt phages GIL16c and Bam35c [19]. These discoveries provide insight into the evolution of the phages as well as the hosts.

In the present study, a novel lysogenic phage named BtCS33, was isolated from Bt strain CS33, which has high toxicity to Lepidoptera*n* and Diptera larvae. This is the first report of a *Siphoviridae* family isolate among the sequenced *B. thuringiensis* phages. The complete genome of phage BtCS33 was sequenced, characterized and compared with other phages that infect the same or closely related species hosts. This is also the first report of a phage isolated from *B. thuringiensis* exhibiting high sequence similarity to *B. cereus* phage Wβ and some of its variants. But, the host range is quite different, for phage Wβ and it's variants can infect Ba and BtCS33 can't infect Ba. This study may provide more information about evolutionary relationship among these *Bacillus* species.

## Results

### Isolation and morphology of the bacteriophage

**Figure 1:**
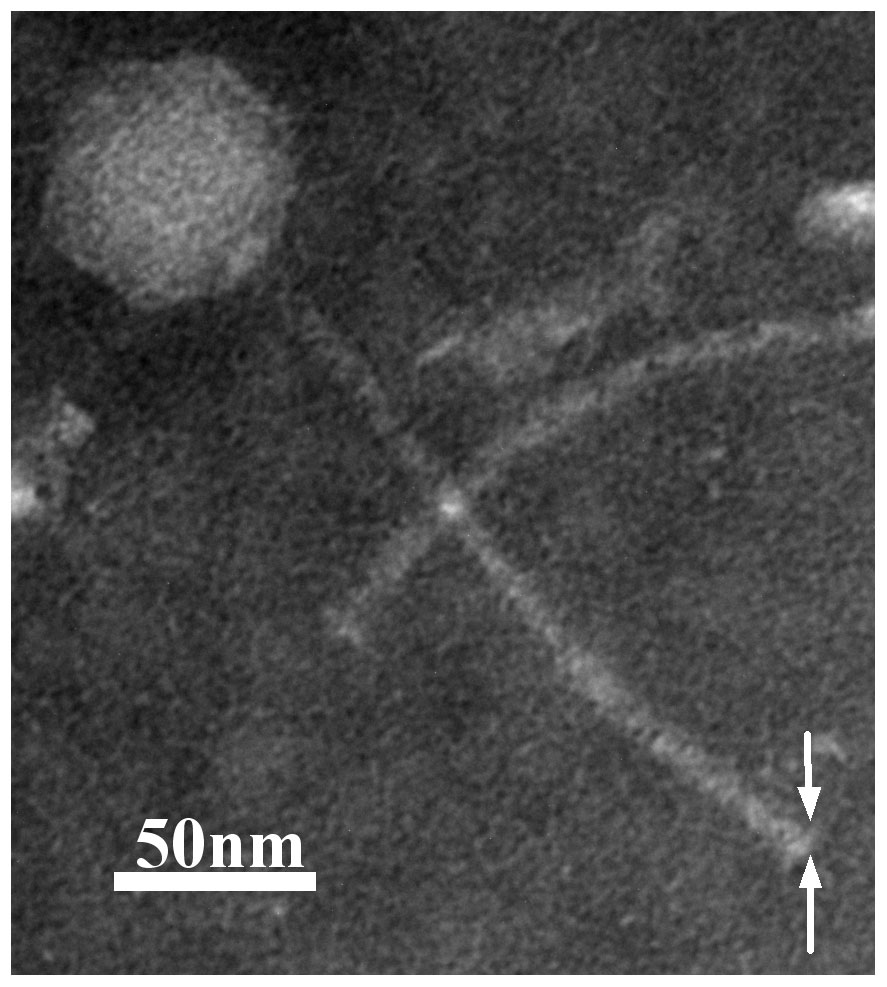
Morphology of phage BtCS33 particles under TEM. The virion was negatively stained with 2% potassium phosphotungstate. The white arrows indicate the putative tail fiber structure.

A bacteriophage designated BtCS33 was isolated from *B. thuringiensis* subsp. *kurstaki* strain CS33, which has high toxicity to Lepidoptera larvae. Transmission electron microscopy showed that BtCS33 had an icosahedral head (61 nm×67 nm) and a long tail (204 nm×5.7 nm) (Figure 1). The phage BtCS33 was similar in shape to *B. cereus* phage Wβ [18] and its variant, Gamma [20], and was considered to be *Siphoviridae*.

### Host range of phage BtCS33

To investigate the sensitivity of *Bacillus* strains to BtCS33, 78 *B. thuringiensis* strains, 4 *B. sphaericus* strains, 2 *B. anthracis* strains, and 1 *B.cereus* strain were tested. The tests showed that only two *B. thuringiensis* strains, CS33 (H-serotype 3) and C-3 (H-serotype 3), were sensitive to phage BtCS33, indicating that BtCS33 had a narrow host range.

### Overview of phage BtCS33 genome

**Figure 2:**
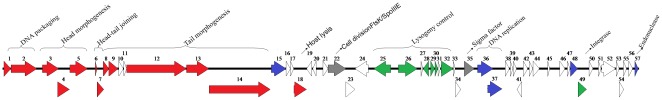
Genome organization of phage BtCS33. The schematic represents the whole genome with the ORFs numbered from left to right. Different colors indicate three regions: the “late” region (red color), the “lysogeny-lysis control” region (green color) and the “early” region (blue color). Gray indicates the genes involved in host cell sporulation. Genes with unknown functions are indicated by white. The orientations of the arrows indicate the direction of transcription.

The complete genome of phage BtCS33 was 41,992 bp in length with an overall G+C content of 35.22%, about the same as the 35.29% G+C% content of the geome of *B. thuringiensis* subsp. *kurstaki* strain BMB171 (Genebank accession number NC_014171). Exonuclease III treatment (data not show) indicated the physical structure of the genome DNA was linear. Sequence analysis revealed 57 putative ORFs (Table S1). The combined length of all ORFs covered 35,432 bp, about 84.4% of the whole genome. The average length of each ORF was 737 bp with ATG as the main start codon, except for ORF1 which had a GTG start codon. Among the 57 ORFs, 51 were transcribed forward, whereas 6 were transcribed in the opposite direction (Figure 2). The genes transcribed rearward were ORF19, ORF20, ORF24, ORF25, ORF28 and ORF41. Furthermore, the start codons of 13 ORFs (22.8% of the total) overlapped with the stop codon of the previous gene. Several promoters of the σ^70^ family were identified by using Bprom (data not show). No ORFs encoding tRNA were found by analysis with tRNAscan-SE 1.21.

On the basis of homology comparisons, 26 ORFs were assigned putative functions. As in other sequenced phages, the major functions were organized in gene clusters (Figure 2). The BtCS33 genome contained three main clusters: the “late” region (encoding structural, assembly, DNA packaging and lysis proteins), the “lysogeny-lysis control” region (encoding proteins for controlling the lysogeny-lysis process) and the “early” region (encoding proteins for phage DNA replication, recombination and modification).

### The structural and lysis module

This module corresponded to the “late” region, encoding proteins for phage structure, assembly and DNA packaging. The module could be divided into five submodules, representing DNA packaging, head morphogenesis, head-tail joining and tail morphogenesis functions (Figure 2). The module included proteins GP1 and GP2 encoded by ORF1 and ORF2 that homologous with (or that had domains typical of) the small and large subunits of terminase. The module also included ORFs encoding portal protein (GP3, gene product of ORF3), major capsid protein (GP5), major tail protein (GP9), tail tape measure protein (GP12) and tail fiber protein (GP13). Comparing the amino acid sequence of GP18 with the CDD database revealed similarity to the GH25-PlyB-like protein, which is a bacteriophage endolysin with potential lytic activity toward *B.anthracis* [21]. Endolysins are produced by phages at the end of their life cycle and participate in lysing the bacterial cell wall to release the newly formed virions [22].

### The lysogeny control module

This module controls the lysogeny-lysis process of the phage. Phage BtCS33 is a lysogenic phage that begins the lysis cycle spontaneously. At least seven putative ORFs were involved in the lysogeny control module. Besides ORF49, six other ORFs were physically related to each other but were transcribed in different directions (Figure 2). ORF49 was predicted to encode integrase, a DNA breaking-joining enzyme that catalyzes site-specific integration of the DNA [23], [24]. Other genes of this module associated with the lysis/lysogeny switch function are are commonly present in temperate *Siphoviridae* phages. GP28 displayed homology to the Cro/CI family proteins, which contain a classical helix-turn-helix domain and can be assigned to the XRE family of transcriptional regulators. GP30 showed identity to many DNA binding proteins and might represent a Cro analogue. The closest BLASTP match for GP32 was an antirepressor that can inactivate the CI repressor [25], [26].

### The DNA replication and recombinant module

This module corresponded to the “early” region, encoding proteins for phage DNA replication, recombination and modification processes. At least three gene products (GP36, GP37, and GP57) of phage BtCS33 were involved in the replication and recombinant process. The amino acid sequence of GP36 was exactly matched with the replication protein O from *Bacillus* phage lambda Ba01. ORF37 was predicted to encode a DNA replication protein (GP37) with an ATP/GTP binding P-loop motif. GP57 demonstrated similarity with various HNH endonucleases from phage and bacteria. HNH endonuclesases belongs to the homing endonuclease family and confers the mobility or duplication of their coding and flanking sequences by a recombination-based process [27], [28]. Genes encoding for HNH endonucleases in phage genomes are considered to be analogous to insertion or transposon elements in bacterial genomes [29].

### The genes associated with host cell sporulation

The genome of BtCS33 contained two genes of particular interest, ORF35 and ORF22. The genes encode proteins with 83% and 100% amino acid sequence similarity, respectively, to proteins from *Bacillus thuringiensis* subsp. *kurstaki* strain T03a001. ORF35 was predicted to encode an RNA polymerase sigma factor (σ^70^) with about 23% similarity to sigmaF from *Bacillus thuringiensis* 97-27. SigmaF can direct expression of sporulation genes in *Bacillus* strains. Many phage RNA polymerase σ factors have been analyzed that can drive phenotypic alteration of the host bacteria [30]. Therefore, we inferred that ORF35 has the ability to regulate the host gene transcription during the sporulation phase [16], [18], [31]. ORF22 encoded a FtsK/SpoIIIE ATPase, an enzyme that plays important roles in intercellular chromosomal DNA translocation and asymmetric division during sporulation [32], [33]. In *B. subtilis*, FtsK/SpoIIIE ATPase was also involved in transferring missegregated DNA during vegetative growth [34]. The cross talk between the phage BtCS33 and the host cell may influence the division of the host cell as well as the lytic life cycle of the phage. Confirming the exact functions of the ORF35 and ORF22 proteins in the phage requires further investigation.

### Comparison of phage BtCS33 and *B. cereus* phage Wβ genomes

**Figure 3:**
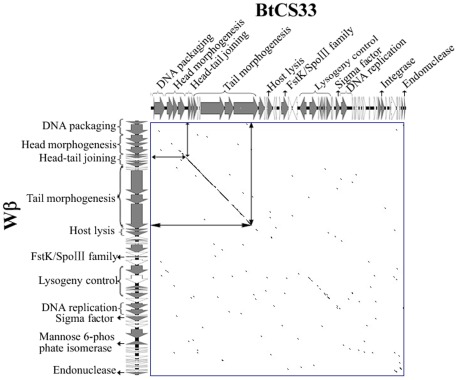
Dot plot alignment of genomes from *B. thuringiensis* phage BtCS33 and *B. cereus* phage Wbeta (Wβ). Arrows indicate the start and the end positions of the most similar fragments on both genomes, corresponding to the dot plot. Both genomes were ranked in the orders of “late” region, the “lysogeney control” region and the “early” region.

**Figure 4:**
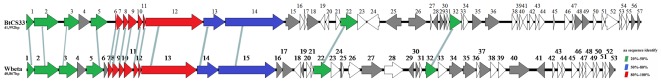
Alignment of the proteomes of *B. cereus* phage Wbeta (Wβ) and *B. thuringiensis* phage BtCS33. The putative proteins are numbered and different color arrows show the levels of amino acid identity: green indicates 20%–50%; blue, 50%–80%; and red, 80%–100%.

BLASTN analysis of the genome ofphage BtCS33 revealed the closest matches to be the genomes of *B. cereus* phage Wβ (NC_007734), and some variants of phage Wβ, such as phage Gamma (NC_007458), Cherry (NC_007457), and Fah (NC_007814). A dot plot of the BtCS33 and Wβ genomes (Figure 3) showed high co-linearity mainly in the “late” regions of each genome encoding the head-tail joining proteins and tail morphogenesis proteins. The five most similar fragments in their “late” regions were shown in Table 1. Raymond Schuch *et al*. reported high sequence similarity between phage Gamma and Wβ [17], [18]. BtCS33 and Gamma also have co-linearity in the “late” region of both genomes (data not shown). Pairwise alignment of the proteomes of phage BtCS33 and phage Wβ also revealed similar genome organization and high homology of ORFs in the “late” region (Figure 4). The similarity between proteins encoded by ORFs 6 to 12 in BtCS33 and ORFs 7to 13 in Wβ was more than 80%, while proteins encoded by ORFs 13 and 14 in BtCS33 and ORFs 14 and 15 in Wβ was more than 50% similar.

**Table 1:** The five most similar nucleotide sequence fragments between genomes of phage BtCS33 and phage Gamma.

BtCS33	Gamma	Length(bp) (BtCS33/Gamma)	Similarity (%)
Position (From..to)	Position (From..to)		
5900..10207	5662..9980	4308/4319	84
10566..12713	10266..12413	2148/2148	82
13433..15447	13143..15160	2015/2018	83
16078..16470	15917..16309	393/393	87
40456..40491	39957..39992	36/36	100
20013..20050	18487..18524	38/38	97

### Phylogenetic tree analysis of phage BtCS33

**Figure 5:**
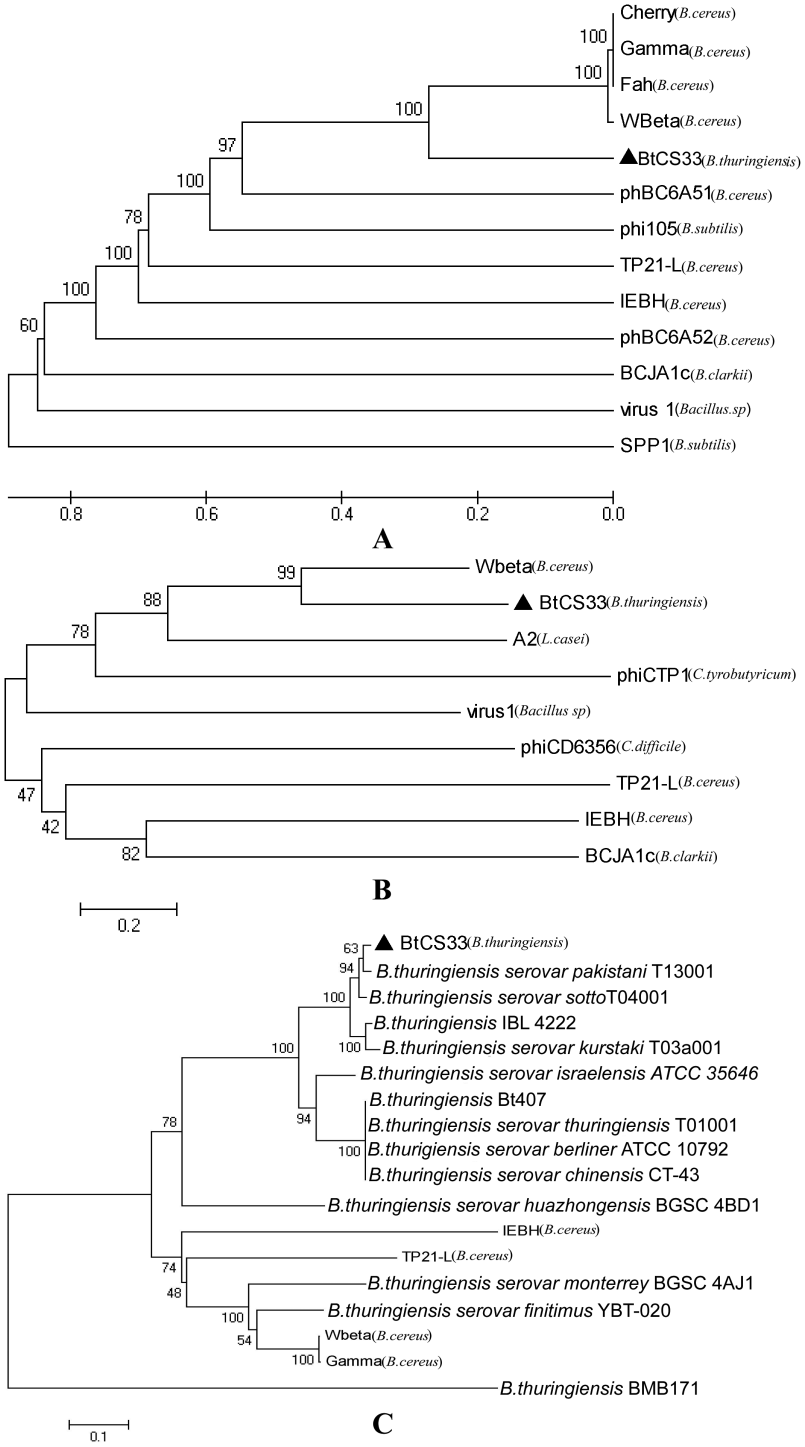
Phylogenetic tree constructed based on the complete geneome and the structural proteins. (A) Phylogenetic tree constructed from the complete genome sequences of *Bacillus* spp. phages using a ClustalW alignment and the UPGMA (unweighted pair group method with arithmetic mean) with bootstrap analysis (1,000 replicates). (B) and (C) Phylogenetic trees constructed by using the neighbor-joining method and bootstrap analysis (1,000 replicates) of a Muscle alignment [35] of amino acid sequences of the major capsid proteins and the tail fiber proteins, respectively. The host species is indicated after the name of each phage. ▴ represented the phage isolated in this study. The numbers on the lines indicated the supporting rates. The strains indicated the origin of the tail fiber proteins in (C). The numbers on the lines indicate the branch support.

To analyze the evolutionary relationship between phage BtCS33 and other phages originating from *Bacillus species*, a phylogenetic tree based on the complete genome sequences from 13 *Bacillus Siphoviridae* phages was constructed (Figure 5A). BtCS33 together with *B. cereus* phages Wβ, Fah, Cherry, Gamma clustered together, while some other phages from *Bacillus* were clustered into different subgroups. Another phylogenetic tree was constructed based on amino acid sequences of the major capsid proteins, which are relatively conserved in phage genome of the *Siphoviridae* family (Figure 5B). Notably, these two phylogenetic trees were in perfect accord showing the same cluster of phage Wβ and BtCS33. The collective results above indicated that, among the sequenced phages from *B. thuringiensis*, BtCS33 was the first reported member of the *Siphoviridae* family and was closely releat to the sequenced phages from Bc.

The tail fiber proteins of phages were reported to be essential for cell wall receptor recognition and binding, which can determine the host specificity of the phages [18]. Because of the extremely narrow host range of phage BtCS33, a phylogenetic tree based on amino acid sequences of the tail fiber proteins was constructed. In total, sequences from 5 *Bacillus Siphoviridae* phages and 13 *B. thuringiensis* prophages were used. The tree showed that the tail fiber protein of BtCS33 was closely related to the tail fiber proteins from many *B. thuringiensis* prophages, but was distantly releated to the tail fiber protein of Wβ (Figure 5C).

## Discussion

In this study, a novel phage BtCS33 isolated from *B. thuringiensis kurstaki* strain CS33 was sequenced and characterized. The complete genome of BtCS33 exhibited some interesting features.

First, phage BtCS33 is different from other sequenced *B. thuringiensis* phages. Until now, five phages originating from *B. thuringiensis* had been sequenced. Three of them (GIL16c, GIL01 and Bam35c) belonged to the *Tectiviral* family and were clustered in the same lineage; Phage 0305-phi8-36, an atypical *Myovirus*, possibly represented a novel, ancient phage lineage, and MZTP02, a *Podoviridae* phage belonging to the phi29 family, was clustered with other lineages. So BtCS33 was the first reported member of the *Siphoviridae* family from *B. thuringiensis*. It clustered with phages from Bc and should be considered a Wβ-group phage (Figure 5).

Second, it was reported that the recognition between phage and host is mainly determined by the phage tail fiber; mutations in the tail fiber gene change the infective activity [18], [36]. Although BtCS33 and Wβ had similar genome organizations and high sequence identify in the structural proteins, they only shared 65% amino acid sequence identity in the tail fiber proteins and the host ranges were different. The low identity of the tail fiber proteins between BtCS33 and Wβ might be the reason for their different host ranges. The tail fiber protein of BtCS33 had high homology with proteins from several *B. thuringiensis* prophages (Figure 5C), but BtCS33 had an extremely narrow host range and did not infect 77 tested *B. thuringiensis* strains except of its host (CS33) and C-3. Therefore, the cause of the narrow host specificity of BtCS33 among Bt strains is still unclear. The tail fiber proteins may not be the key or single factor determining the host specificity of BtCS33. Efforts to understand the cause of the narrow host range of BtCS33 are ongoing.

Third, new evidence for the evolution of *Bacillus* phage was found. Two genes, named ORF22 and ORF35 encoding FtsK/SpoIIIE ATPase and RNA polymerase factor, respectively, were found in the genome of BtCS33, and had high similarty to the correspondence genes in the geneome of *B. thuringiensis* subsp. *kurstaki* strain T03a001, which belongs to the same subspecies as the host of BtCS33. Furthermore, genes with similar functions were also found in *B. cereus* phage Wβ, phage Gamma and some other Gamma isolates [16]–[18]. The phage-encoded RNA polymerase sigma factor regulates vegetative growth as well as sporulation of the host bacteria. This represents a kind of cross-talk between the phage and host, and has been reported previously [31]. Obtaining genes involved in sporulation may be a common phenomenon for phages from sporulating bacteria. The exact function of the putative sporulation genes in the genome of BtCS33 will be further studied. From the combined evidences of host-related genes and the phylogenetic trees, it can be inferred that these phages may have a common distant ancestor and BtCS33 should be considered as a Wβ-group phage.

Fouth, from a different perspective, the similarity of these phages was evidence of the evolution of the host bacterial species. Classifying of *B. anthracis*, *B. thuringiensis* and *B. cereus* as a single species, *Bacillus cereus sensu lato*, remains a matter of debate [37], [38]. The genome similarity of these phages provides more evidence for classifying the three *Bacillus* species as a single species.

## Materials and Methods

### Culture media and bacterial strains

*Bacillus thuringiensis* subsp. *kurstaki* CS33 was isolated in our lab and is highly toxic to insects larvae of Lepidopteran and Dipteran species.

### Isolation and propagation of the phage

Bacteriophage was isolated from *B. thuringiensis* strain CS33 according to the modified method described by Carey-Smith *et al* [39]. Bt strain CS33 was incubated on a nutrient plate to form plaques. One plaque was picked and suspended in 1 ml SM buffer [0.58% (w/v) NaCl, 0.2% (w/v) MgSO_4_, 50 mM Tris-HCl, pH 7.5] as a bacteriophage suspension. To get pure phage, 100 µl of phage suspension was mixed with 300 µl of CS33 culture during exponential phase growth (OD_600_ about 1.0), and the mixture was added into 5 ml of molten semisolid medium (at about 45°C). After thoroughly mixing, the semisolid medium containing bacteria and phages was poured onto a solid medium plate as an overlay and incubated at 30°C overnight (12–16 hours). After plaques formed on the upper medium, one plaque was picked, and the process above was repeated at least five times until homogeneous plaques formed. Finally, the pure bacteriophage was harvested and designated BtCS33.

Propagation of the bacteriophage was performed by using the method as described above, except a bacteriophage suspension at a titer of about 10^6^ PFU/ml was used. After incubating the suspension at 30°C overnight, 5 ml of SM buffer was added onto each plate and left at 4°C at least 4 h with morderate rotation. Then the suspension was recovered and centrifuged at 8,000 g at 4°C for 10 minutes. After the supernatant was filtrated through a 0.22 µm sterile filter, the concentrated phage preparation was stored at 4°C for use.

### Observation of bacteriophages by electron microscopy

A 5 ml aliquot of SM buffer containing 0.01% of gelatin was added onto the bacterium-phage plate, the plate was morderately rotated at 4°C for 2 h, and then the suspension was collected. After centrifugation for 10 min at 8,000 g at 4°C, the supernatant was immediately deposited on cuprum grids with carbon-coated Formvar films, and stained with 2% potassium phosphotungstate (PT, pH 7.2) [7], [40]. After the film was dried in air, it was observed by TEM (HITACHI H-7000FA transmission electron microscope) at an acceleration voltage of 100 kV.

### Investigation of host range

The host range of BtCS33 was investigated using the method described above for the isolation of the phage. A suspension of bacteriophage at about 10^6^ PFU/mL was used to infect the tested *Bacillus* spp. strains. After incubation overnight, plaques on the bacterial plates were observed. These experiments were repeated for three times. In total, 79 *B. thuringiensis* strains including 21 Bt reference strains and 58 isolates belonging to 17 of H-serotypes, 4 *B. sphaericus* (Bs) strains (2 reference strains and 2 isolates), 1 *B. cereus* strain and 2 *B. anthracis* (Ba) strains were tested. *B. anthracis* strains were provided by Dr. Yuan in Wuhan Institute of Virology, Chinese Academy of Sciences, and pXO1 and pXO2 were eliminated in the two *B. anthracis* strains, respectively. Bt and Bs strains tested were kept by our lab.

### Extraction of phage DNA

DNA extraction from phage particles was performed according to the method described by Santos with modifications [41]. Each milliliter of phage suspension was treated with 6 units/ml of DNase and 20 µg/ml of RNase at 37°C for 30 min, 20 µl of a 2 M solution of ZnCl_2_ was added and the phage suspension was incubated at 37°C for 5 min. After centrifugation for 1 min at 10,000 g, the supernatant was removed and the pellet was suspended in 500 µl TES buffer (0.1 M Tris/HCl, pH 8.0; 0.1 M EDTA; 0.3% SDS) and incubated at 60°C for 15 min. After incubation with 20 µl proteinase K (20 mg/ml) at 37°C for 90 min, 60 µl of 3 M potassium acetate solution (pH 5.2) was added to the suspension and completely mixed; then, the mixture was kept on ice for 15 min. The mixture treated with phenol/chloroform/isoamyl alcohol (25∶24∶1, v/v) twice, and then with chloroform/isoamyl alcohol (24∶1, v/v) once. Phage DNA was precipitated with an equal volume of isopropanol washed with 70% ethanol twice, and dissolved in 10 µl distilled water. The DNA was checked by 0.6% agarose-gel electrophoresis.

### Proteinase K and exonuclease III treatment of the BtCS33 genome

DNA preparations of BtCS33, which were prepared proteinase K pretreatment, were incubated with variable amounts of proteinase K (0.01 and 0.1 mg/ml) for 4 h at 37°C [11]. BtCS33 DNA preparations were also treatment with exonuclease III according to the manufacturers' instructions. The effect of the treatment was analyzed by running the BtCS33 DNA preparation on a 0.6% agarose gel

### Genomic DNA sequence and bioinformatics analysis

Genomic DNA sequencing was performed by BGI Co. (Beijing, China) with a shotgun sequencing method and the genome was assembled with phrap version 1.080812. Each base had at least five-fold coverage. Open reading frames (ORFs) were predicted with FGENE SV software (http://linux1.softberry.com/berry.phtml?topic=virus&group=programs&subgroup=gfindv) and by visual inspection. ATG, GTG and TTG were used as possible start codons, and 30 codons were taken as a threshold for the ORFs. The BLAST (http://www.ncbi.nlm.nih.gov/blast/Blast.cgi) was used to search for homologous proteins, and the function of each ORF was compared in the CDD database at NCBI (http://www.ncbi.nlm.nih.gov/Structure/cdd/cdd.shtml). To search for putative tRNA-encoding genes, tRNAscan-SE 1.21 (http://selab.janelia.org/tRNAscan-SE/) was used. Promoter sequences in the σ^70^ family were identified by using Bprom (http://linux1.softberry.com/berry.phtml). Dot plot analysis was performed using Nucleic Acid Dot Plots (http://www.vivo.colostate.edu/molkit/dnadot/index.html) with a window size of 13 and a mismatch limit of 0.

### Nucleotide sequence accession number

The complete nucleotide sequence of BtCS33 was submitted to GenBank under accession number is JN191664.

## Supporting Information

Table S1 General features of the predicted proteins from BtCS33 genome.
(DOC)
