# Supplementary material for: Genome Characteristics of a Novel Phage from Bacillus thuringiensis Showing High Similarity with Phage from Bacillus cereus
Source: PLoS One. 2012 May 23;7(5):e37557. doi: 10.1371/journal.pone.0037557 (PMC3359378; doi:10.1371/journal.pone.0037557)
Supplement: Table S1 — General features of the predicted proteins from BtCS33 genome. (DOC) [file pone.0037557.s001.doc]

Table S1. General features of the predicted proteins from BtCS33 genome

| ORF  Name | Position(nt) | ORF  length(aa) | Size  (kDa[pI]) | Closest hit  (e value) | Accession no.  of closest hit | The best match in phages (e value) | Predictive  function |
| --- | --- | --- | --- | --- | --- | --- | --- |
|  | From...To |  |  |  |  |  |  |
| 1 | 62..565 | 167 | 18.9(9.10) | *B.thuringiensis* ATCC35646(5e-13) | ZP_00742397 | *Lactobacillus* phage Sal1(2e-20) | Terminase small subunit |
| 2 | 567..2261 | 564 | 65.1(5.73) | *B.thuringiensis* T03a001 (0.0) | ZP_04117837 | *Geobacillus* virus E2(0.0) | Terminase large subunit |
| 3 | 2627..3703 | 358 | 40.7(6.10) | *B.thuringiensis* T03a001 (0.0) | ZP_04117838 | *Bacillus* phage phi105 (1e-77) | Portal protein |
| 4 | 3621..4400 | 259 | 29.1(4.74) | *B. thuringiensis* ATCC35646(1e-129) | ZP_00743661 | *Lactobacillus* phage Sal1(9e-40) | Endopeptidase clp proteolytic subunit |
| 5 | 4438..5604 | 388 | 43.3(5.00) | *B. thuringiensis* ATCC35646(0.0) | ZP_00743660 | *Bacillus* phage phi105(3e-85) | Major caspid protein |
| 6 | 6118..6222 | 34 | 4.0(9.87) | *B.thuringiensis* IBL 4222(2e-11) | ZP_04068672 | *Bacillus* phage Gamma, Cherry, Wβ, Fah(6e-09) | Head-tail adaptor |
| 7 | 6215..6652 | 145 | 16.2(9.36) | *B*.*thuringiensis* BGSC 4AJ1(4e-78) | ZP_04112614 | *Bacillus* phage Gamma, Cherry, Wβ,Fah(8e-62) | Head-tail joining protein |
| 8 | 6649..7008 | 119 | 13.8(5.41) | *B.cereus* B4264(7e-61) | YP_002367337 | *Bacillus* phage Gamma, Cherry, Wβ, Fah(1e-56) | Structure protein |
| 9 | 7009..7614 | 201 | 22.4(5.25) | *B.thuringiensis* T03a001 (4e-109) | ZP_04118224 | *Bacillus* phage Gamma, Cherry, Wβ, Fah(2e-93) | Major tail protein |
| 10 | 7661..7978 | 105 | 11.6(4.64) | *B.thuringiensis* T13001 (3e-55) | ZP_04118223 | *Bacillus* phage Gamma, Cherry, Wβ, Fah(1e-45) | Hypothetical protein |
| 11 | 8008..8184 | 58 | 7.1(5.32) | *B.thuringiensis* T13001 (2e-22) | ZP_04124015 | *Bacillus* phage Gamma, Cherry, Wβ, Fah(1e-17) | Hypothetical protein |
| 12 | 8196..12122 | 1308 | 142.7(6.79) | *B.cereus* B4264(0.0) | YP_002367341 | *Bacillus* phage Gamma, Cherry, Wβ, Fah(0.0) | Tail tape measure protein |
| 13 | 12134..13618 | 494 | 56.8(5.94) | *B.thuringiensis* T04001 (0.0) | ZP_04127931 | *Bacillus* phage Gamma, Cherry, Wβ, Fah(0.0) | Tail fiber protein |
| 14 | 13615..17640 | 1341 | 151.5(5.59) | *B. thuringiensis* ATCC35646 (0.0) | ZP_00741743 | *Bacillus* phage Fah(0.0) | Minor structure protein |
| 15 | 17728..18696 | 322 | 38.0(9.19) | *B.thuringiensis* T03a001 (0.0) | ZP_04113280 | *Bacillus* phage  11143(3e-179) | Site-specific recombinase |
| 16 | 18710..18991 | 93 | 10.4(5.87) | *B.thuringiensis* T03a001 (4e-45) | ZP_04113281 | *Bacillus* phage  11143(1e-29) | Hypothetical protein |
| 17 | 18994..19206 | 70 | 8.0(6.55) | *B.thuringiensis* T03a001 (3e-33) | ZP_04113282 | *Bacillus* phage  11143(4e-30) | Hypothetical protein |
| 18 | 19206..20024 | 272 | 31.1(9.61) | *B.thuringiensis* T03a001 (8e-160) | ZP_04113283 | *Bacillus* phage  11143(2e-115) | N-acetylmuramoyl-L-alanine amidase |
| 19 | -/(20065..20394) | 109 | 12.3(9.6) | *B.thuringiensis* T03a001 (4e-56) | ZP_04113284 | *Bacillus* phage phBC6A51(1e-19) | Hypothetical protein |
| 20 | -/(20463..20684) | 73 | 8.5(10.06) | *B.thuringiensis* T03a001 (5e-34) | ZP_04113285 | *Bacillus* phage IEBH(5e-06) | Hypothetical protein |
| 21 | 21147..21467 | 106 | 12.0(9.93) | *B.thuringiensis* T03a001 (7e-55) | ZP_04113286 |  | Hypothetical protein |
| 22 | 21478..22644 | 388 | 45.0(8.61) | *B.thuringiensis* T03a001 (0.0) | ZP_04113287 | *Geobacillus* phage GBSV1(2e-72) | Cell division FtsK/SpoIIIE ATPase |
| 23 | 22634..23242 | 202 | 24.1(9.65) | *B.thuringiensis* CT-43 (6e-112) | AEA14343 | *Geobacillus* phage GBSV1(2e-45) | Hypothetical protein |
| 24 | -/(23247..24128) | 293 | 34.1（9.76） | *B.thuringiensis* T03a001 (2e-170) | ZP_04113289 | *Bacillus* phage Gamma, Cherry, Wβ, Fah(4e-04) | Hypothetical cytosolic protein |
| 25 | -(24502..25602) | 366 | 42.3(9.74) | *B.thuringiensis*  ATCC35646(2e-172) | ZP_00741791 | *Clostridium* phage phi3626(2e-149) | DNA integration/ recombine ation/inversion protein |
| 26 | 26109..27359 | 416 | 48.6(4.86) | *B.thuringiensis* T03a001 (0.0) | ZP_04118234 | *Bacillus* phage Gamma, Cherry, Wβ, Fah(1e-104) | HTH transcriptional regulator |
| 27 | 27600..27731 | 43 | 4.9(4.03) | *B.thuringiensis* T03a001 (1e-14) | ZP_04118233 |  | Hypothetical protein |
| 28 | -/(27759..28103) | 114 | 13.3(8.8) | *B.thuringiensis* T03a001 (1e-59) | ZP_04118232 | *Bacillus* phage Gamma, Cherry, Wβ, Fah(5e-14) | Transcription regulator  Putative Cro/CI family |
| 29 | 28252..28488 | 78 | 9.3(6.73) | *B.thuringiensis* T03a001 (2e-38) | ZP_04118231 | *Listeria* phage A500(0.51) | Transcription regulator |
| 30 | 28521..28709 | 62 | 7.3(9.65) | *B.thuringiensis* T03a001 (5e-28) | ZP_04118230 | *Staphylococcus* phage 92(5e-04) | Transcription regulator |
| 31 | 28734..28889 | 51 | 6.0(10.37) | B.thuringiensis T13001 (1e-20) | ZP_04123682 | *B.thuringiensis* phage MZTP02 (4e-16) | Hypothetical protein |
| 32 | 28935..29714 | 259 | 29.6(7.81) | *B.thuringiensis* IBL4222 (2e-147) | ZP_04068725 | *Bacillus* phage Gamma, Cherry, Wβ, Fah(3e-55) | Antirepressor |
| 33 | 29739..29855 | 38 | 4.3(4.51) | *B.thuringiensis* T03a001 (8e-13) | ZP_04117906 |  | Hypothetical protein |
| 34 | 29852..30190 | 112 | 13.1(8.93) | *B.thuringiensis* T03a001 (7e-53) | ZP_04117905 | *Bacillus* virus1 (3.3) | Hypothetical protein |
| 35 | 30465..31112 | 215 | 25.1(6.72) | B.thuringiensis IBL 4222(2e-116) | ZP_04068728 |  | RNA polymerase σ factor |
| 36 | 31336..32352 | 338 | 40.2(8.45) | *B.thuringiensis* ATCC35646(3e-165) | ZP_00742078 | *Bacillus* phage lambda Ba01(2e-38) | Replication protein O |
| 37 | 32186..33127 | 313 | 36.5(8.97) | *B.thuringiensis* ATCC35646 (5e-157) | ZP_00742079 | *Bacillus* virus1 (2e-13) | DNA replication protein-like protein |
| 38 | 33169..33435 | 88 | 10.4(4.89) | *B.thuringiensis* ATCC35646 (2e-41) | ZP_00742080 | *Bacillus* phage IEBH(2e-20) | Phage protein |
| 39 | 33507..33671 | 54 | 6.3(9.7) | *B.thuringiensis* ATCC35646 (3e-23) | ZP_00742081 | *Bacillus* phage 11143(0.008) | Phage protein |
| 40 | 33689..33904 | 71 | 8.4(7.76) | *B.thuringiensis* ATCC35646 (3e-35) | ZP_00742082 | *Staphylococcus* phage phi12 (0.003) | Hypothetical protein |
| 41 | -/(33901..34200) | 99 | 11.6(5.38) | *B.thuringiensis* IBL4222 (6e-50) | ZP_04063415 | *Lactobacillus* prophage Lj928 (1.1) | Hypothetical protein |
| 42 | 34351..34650 | 99 | 11.6(5.06) | *B.thuringiensis* T03a001 (1e-50) | ZP_04117948 | *Bacillus* phage 11143(1e-40) | Hypothetical protein |
| 43 | 34750..34980 | 76 | 8.7(9.41) | *B.thuringiensis* T04001 (1e-17) | ZP_04130039 |  | Hypothetical protein |
| 44 | 35004..35411 | 135 | 15.6(4.99) | *B.thuringiensis* T03a001 (2e-73) | ZP_04117949 | Bacteriophage A118(1.8) | Hypothetical protein |
| 45 | 35968..36087 | 39 | 4.7(7.76) | *B.thuringiensis* T03a001 (9e-14) | ZP_04117950 |  | Hypothetical protein |
| 46 | 36766..37101 | 111 | 13.3(5.38) | *B.thuringiensis* ATCC35646 7e-57) | ZP_00742090 |  | Hypothetical protein |
| 47 | 37267..37431 | 54 | 6.8(10.24) | *B.thuringiensis* T03a001 (9e-22) | ZP_04117916 | *Bacillus* phage phBC6A52(2e-06) | Phage protein |
| 48 | 37459..37944 | 161 | 18.8(7.71) | *B.thuringiensis* T03a001 (7e-90) | ZP_04117915 | *Bacillus* phage phBC6A52(1e-72) | Transcription regulator |
| 49 | 37941..38483 | 180 | 21.0(9.86) | *B.cereus* AH1273(3e-98) | ZP_04174359 | *Bacillus* phage  phBC6A52(7e-93) | Integrase |
| 50 | 38690..38935 | 81 | 9.7(4.73) | *B.thuringiensis* T03a001 (2e-39) | ZP_04118185 | *Bacillus* phage phBC6A52(2e-13) | Hypothetical protein |
| 51 | 39307..39489 | 60 | 6.8(4.31) | *B.thuringiensis* T03a001 (5e-26) | ZP_04118184 |  | Hypothetical protein |
| 52 | 39538..40437 | 299 | 34.9(5.5) | *B.thuringiensis* T03a001 (1e-171) | ZP_04118183 |  | Hypothetical protein |
| 53 | 40486..40671 | 61 | 6.9(5.68) | *B.thuringiensis* T03a001 (4e-26) | ZP_04118182 | *Bacillus* phage phBC6A51(6e-15) | Hypothetical protein |
| 54 | 40658..40939 | 93 | 10.4(5.88) | *B.thuringiensis* T13001 (4e-30) | ZP_04123704 | *Bacillus* phage Gamma, Cherry, Wβ, Fah(1e-16) | Hypothetical protein |
| 55 | 40956..41168 | 70 | 8.1(6.22) | *B.thuringiensis*  T03a001 (7e-32) | ZP_04118180 | *Bacillus* phage IEBH(6e-14) | Hypothetical protein |
| 56 | 41304..41558 | 84 | 10.0(9.02) | *B.cereus* B4264 (2e-42) | YP_002367327 | *Bacillus* phage Gamma, Cherry, Wβ, Fah(9e-33) | Conserved phage protein |
| 57 | 41719..41925 | 68 | 8.1(9.39) | *B.cereus* B4264(6e-32) | YP_002367328 | *Bacillus* phage Gamma, Cherry, Wβ, Fah(1e-12) | HNH endonuclease |
